# The Fundamental Principles of Fracture Treatment

**Published:** 1942

**Authors:** K. H. Pridie

**Affiliations:** Honorary Surgeon-in-charge of Bristol Royal Hospital E.M.S. Fracture Unit; Consulting Surgeon, Bridgwater Hospital


### THE FUNDAMENTAL PRINCIPLES OF FRACTURE TREATMENT.

BY

K. H. PRIDIE, F.R.C.S.

*Honorary Surgeon-in-charge of Bristol Royal Hospital E.M.S. Fracture Unit;*
*Consulting Surgeon, Bridgwater Hospital.*

---

ALTHOUGH the methods of reduction and fixation of fractures have been constantly changing since the days of Hippocrates the method of healing has never changed, and never will. It takes a very considerable force to fracture a normal bone: If the bone breaks after a trivial accident the fracture is said to be pathological.

Bone shows an exception from the normal healing of other structures which heal by fibrous tissue. A bone repairs itself by bone. The natural stimuli to union after a fracture in a normal bone is thought to be one or both of :

1. Blood set free at the site of fracture.
2. The two fractured bone ends in contact.

Any conditions which interferes with these natural stimuli will delay healing, *e.g.* :

1. *Factors affecting the hæmatoma.*
   (*a*) Small crack and small hæmatoma.
   (*b*) Transverse fracture and little stripping of periosteum, and consequent small hæmatoma.
   (*c*) Open operation and loss of hæmatoma.
   (*d*) Compound fracture and loss of hæmatoma.
   (*e*) Sepsis and loss of hæmatoma.
2. *Factors affecting the contact of bone ends.*
   (*a*) Separation of bone ends by excessive traction.
   (*b*) Muscle interposition.
   On the other hand good apposition of bone ends, where only a small crack has occurred or where reduction is perfect, promotes rapid union.

### THE PATHOLOGY OF HEALING.

There are four stages in the healing of a fracture :

1. Hæmatoma at the site of fracture which in a few days produces a clot.

2. Invasion of the clot by calcium salts. This takes place from the sixth to the twelfth week. The calcified clot forms an external scaffolding which holds the bones firm until the internal callus is formed. When this stage has been reached union is said to have occurred. The bones may still angulate at the site of fracture, but they will not over-ride.

3. The internal callus is formed and the union becomes consolidated. This stage progresses from the sixth week to the sixth month.

4. Re-modelling of the bones. The excess soft external callus is absorbed and the original shape of the bone is re-constituted. If the position is not good, in young people the body has the power to re-model the bone, adding or taking away, and so often making a good result from an apparently bad one. This process starts at about the third month and may continue for one or even two years.

A clear conception of what the healthy body is capable of doing by itself at various ages is essential before a surgeon can assess the necessity for performing an open operation for reduction or fixation.

1*st Age.*—So great a power of regeneration of bone is found in the bones of the new-born infant that fractures do not even need splinting. However bad the X-ray appearance is at the time of the fracture, provided the alignment is corrected, within a year it is impossible to tell which side has been broken.

2*nd Age.*—The young child has the most wonderful powers of regenerating bone shape and it is difficult to obtain a bad result that will be permanent in a child under five years. But whereas nature allows great latitude with the shafts of the bones great care must be taken when the epiphyses are displaced.

3*rd Age.*—In the treatment of fractures of the adolescent there are few difficulties that really worry the fracture surgeon.

4*th Age.*—It is during the age when the individual meets the motor-cycle, the motor-car and the aeroplane that the real troubles begin. It is now that not only are the bones broken but the soft tissues are severely damaged. During this period it is not the twist fractures of the young that are seen, but the transverse fracture, the comminuted fracture, and the multiple fracture. The "exploding" fracture is seen: here the lesion to the bone is often the least important, but there is damage to the skin, muscles, vessels and nerves as well as to bones.

5*th Age.*—Add to this age of excitement the age of confusion, in which a cross-section of all age groups is subjected to the effects of blast and high explosives. Although the damage to the bones is extensive it may fall into the background when compared to the damage to soft tissues.

6*th Age.*—The age of decline where fracture results are not so satisfactory. Healing does not take place so quickly and the powers of adaption and regeneration are poor.

*7th Age.*—The aged. Degenerative changes in the skin, muscle, bone and vessels cast their shadow on the end results of the treatment and demand that it shall be conservative and easy.

All these fundamental facts have to be carefully considered in the treatment of any fracture.

### THE REDUCTION OF FRACTURES.

There are three classes of simple fractures :—

1. A crack of the bone where there is no displacement or instability of the limb as a result of the crack. These fractures require no reduction or fixation.

2. A complete break, where the limb is unstable. There is apposition of the bone ends, and angulation is the only deformity to be corrected.

3. A bone is broken and the ends are separated. The object of treatment is to convert this type to Class 2.

Many fractures which occur in the home or at sport require no reduction as there is no displacement. In all cases loss of alignment is of more serious consequence than the lack of end to end apposition, particularly in the weight-bearing limbs. Perfect end to end apposition where the broken surfaces are replaced with absolute accuracy, serration for serration, is not often possible to obtain by any other method than open operation. Perfect hair-line reduction, however, is not necessary and is certainly not demanded by nature. The healing processes of the body are capable of joining two bones provided there is contact between these two bones. Even over-lap does not produce non-union: in fact it is often an insurance against non-union.

Does slight over-lap lead to disability ? Shortening of under one inch of the lower limb in an adult leads to no serious disability. Shortening of half an inch can hardly be noticed and shortening of a quarter of an inch is of no consequence whatsoever. It is often the attempt to regain the lost length which causes delay in union and permanent disability.

There are many methods of reducing a fracture, but the great majority of fractures can be reduced manually by using longitudinal traction. Angulation or hinging can be used to aid reduction, especially in transverse fractures. When the reduction is completed the limb should measure the correct length and should look similar to its fellow. In spite of the appearance of the X-ray picture, if the limb unites in this position neither the patient nor the surgeon have much to worry about. So often the appearance of the X-rays frightens the inexperienced surgeon into performing open operations when they are not necessary. It is the experience of most fracture surgeons that in the majority of very severe accidents, where there has been a bad compound fracture, the state of the soft parts is such that open operation is absolutely out of the question. In the minor fractures where the soft parts are in a fit condition for open operation, the reduction is usually fairly simple and can be performed manually. It is only in a very few cases, therefore, that open operation is indicated and it should only be undertaken by a surgeon skilled in fracture work.

*Skeletal Traction.*—Skeletal traction should only be used for the reduction of fractures after manual traction and manipulation have failed. Only in a very small percentage of cases should skeletal traction be necessary as it has very definite dangers and must not be undertaken lightly. Skeletal traction is chiefly used as a means of reduction, and rarely as a means of fixation. When the reduction has been performed it is better immediately to remove the wires or pins that have been used and not to leave them in the bone for fear of infection.

Each surgeon develops his own methods : but these are the general rules governing the treatment of fractures by skeletal traction :—

1. Excessive force must not be used. There is no more potent cause of non-union than over-traction.

2. Skeletal traction is more powerful than strapping extension and therefore less weight is needed to produce the same effect on the bone fragments.

3. When reduction has been obtained by traction the reduction should be maintained by a plaster cast.

4. Skeletal traction when used to apply extension on a limb should be reserved for those cases where strapping extension is not possible on account of skin damage or ulceration.

5. Only in very special circumstances should traction pins or wires be left in the bone and incorporated in the plasters.

6. All pins and wires should be immobilized by plaster so that there is no movement of the pin or wire relative to the bone when skeletal traction is being used for any length of time.

### Methods of Immobilization.

Fractures of those parts of the bone from which muscle takes origin or to which muscle is attached appear to unite by means of external callus, and nature provides immobilization by muscle spasm : *e.g.* a fracture of the surgical neck of the humerus will unite without any form of fixation whatsoever and early movements can be instituted by means of exercises. The bones will be kept in contact and in relative immobilization by the local muscles which are in a state of spasm.

Fractures of regions of the bone which are intra-articular, *i.e.* within the capsule of the joint, have no muscle attachment, and the fracture line is bathed in synovial fluid after the fracture has occurred. This type of fracture never unites by external callus. There is no muscle attachment and therefore no local immobilization by the muscles.

Perfect fixation must be obtained in these cases otherwise union will not occur, and the slightest movement will rupture the small capillaries growing from one bone to the other, preventing union. Fractures of the neck of the femur and of the carpal scaphoid are examples of this. In these cases far more rigid immobilization must be aimed at than for a similar fracture of the shaft of the femur or of the radius above the wrist joint.

Fractures of those areas of bone to which muscle is attached and which unite by external callus need splinting to prevent angulation, but the process of union will take place without any form of external splinting or immobilization whatsoever. The best examples of this are fractures of the ribs and clavicle.

When considering the correct method of immobilization for each type of fracture we must consider it from the first principles and during the four stages of healing. It is not always the bone which is damaged. The soft parts may also be injured and the process of treatment must depend on both the bones and the soft parts. The correct treatment for the soft parts is often quite opposite to that of the bones, and therefore a compromise between the two has to be found.

In the more severe fractures the treatment of the soft parts becomes the most important factor and dominates the picture. Consider the treatment of a severe compound fracture of the tibia. The most important factor here is the soft tissue. The wound has to be excised, the devitalized and contaminated tissue excised, and if possible the wound closed again.

If the fracture is simple in type the bone ends have to be reduced as accurately as possible consistent with the circumstances of the case. The bone must then be held by some form of external fixation until union has occurred, and the fracture site must be protected from angulation until consolidation has taken place.

The most common form of fixation to-day is the plaster of Paris cast which acts as an "external bone" and replaces the internal bone until this has satisfactorily healed.

### FIRST-AID TREATMENT OF FRACTURES.

The first-aid treatment of fractures consists of two parts.

1. The treatment of the wound.
   (*a*) The clothing should be cut away from the wound.
   (*b*) Sulphonamide powder with 2 per cent. light magnesium hydroxide added should be blown into the wound.

(c) A sterile first-aid dressing should be applied with plenty of wool and a firm bandage if there is much hæmorrhage. Firm pressure should be used to control hæmorrhage and only if this fails should a tourniquet be applied. 175 years ago John Hunter made the observation "that if he divided the vessels in the thigh of a boar they stopped bleeding before the animal weakened."

2. The treatment of the bone.

(a) Any gross deformity can be corrected by hand. This relieves kinking of the blood vessels and tends to prevent swelling.

(b) A firm bandage such as "Elastocrepe" should be applied over wool, from the extremity of the limb to at least 6 inches above the fracture site. This supports the tissues and prevents swelling.

(c) An adequate back-splint should be applied with two side splints. These splints must extend well beyond the joint above and below the fracture site. A back splint and foot piece from the toes to the knee will do nothing but harm to a fracture of the middle or upper third of the tibia.

(d) The injured part should be kept elevated above the rest of the body, until adequate reduction can be performed and the plaster cast applied.

### X-RAYS IN FRACTURE TREATMENT.

Every case where a fracture is either obvious or suspected should be X-rayed as soon as possible on admission to hospital. The reduction of the fracture should be confirmed by X-ray plates, but only in very rare cases should the fracture be reduced under the X-ray screen on account of the extreme danger of radiation. Protective gloves render the hands almost as useless as the feet for any manipulation. The position of the bone ends should be checked by further X-rays at every change of plaster.

### ANÆSTHESIA IN FRACTURES.

The type of anæsthesia used depends upon the circumstances in which the fracture is to be set and upon the condition of the patient. In order to reduce a fracture easily there must be complete muscular relaxation of the patient. Often in a busy clinic the quickest form of anæsthetic is given and the reduction results in a struggle between the surgeon and the patient's muscles. The worst possible condition under which any fracture can be set is when the patient is rigid and all the muscles are in a state of spasm. A highly skilled anæsthetist is therefore essential if a good reduction of the fracture is to be expected and maintained while the plaster splint is being applied and is drying.

